# Processing Tomato Responses to Plant‐Based Biostimulants Are Modulated by Environmental Conditions

**DOI:** 10.1111/ppl.70450

**Published:** 2025-08-10

**Authors:** Giovanna Marta Fusco, Andrea Burato, Alfonso Pentangelo, Petronia Carillo, Mario Parisi

**Affiliations:** ^1^ Department of Environmental, Biological and Pharmaceutical Sciences and Technologies University of Campania “Luigi Vanvitelli” Caserta Italy; ^2^ School of Agricultural, Forest, Food, and Environmental Sciences (SAFE) University of Basilicata Potenza Italy; ^3^ CREA Research Centre for Vegetable and Ornamental Crops Council for Agricultural Research and Economics Pontecagnano Italy

**Keywords:** amino acids, climate change, plant resilience, protein hydrolysates, *Solanum lycopersicum* L.

## Abstract

Tomatoes ( *Solanum lycopersicum* L.), rich in health‐promoting bioactive compounds, are vital to the economy and culture of Mediterranean regions such as southern Italy. However, open‐field cultivation in these areas faces increasing challenges due to fluctuating environmental conditions, which intensify oxidative stress, accelerate ripening, and compromise yield and quality. Plant‐based protein hydrolysates (PHs) and optimized irrigation practices have emerged as promising strategies to enhance crop resilience. This study assessed the effects of two foliar‐applied biostimulants: MU, a seaweed and plant amino acid‐based formulation, and SR, a potassium‐rich botanical extract. Both were tested on tomatoes (cv. “H1534”) under open‐field conditions in southern Italy (Foggia) over two seasons (2019–2020). Both biostimulants had no significant impact on yield traits or technological quality, whereas year‐to‐year variability markedly influenced outcomes. In 2020, compared with 2019, total yield declined by 45%, and defective fruits rose by 311%.°Brix, polyphenols, lycopene, and sweetness index also decreased significantly (−41%, −18%, −58%, and −14%, respectively), indicating stress conditions. Under these circumstances, MU increased polyphenols (+27%) and enhanced essential (+42%) and branched‐chain amino acids (BCAAs, +63%), while SR also stimulated BCAAs accumulation (+30%). These findings suggest that, under variable open‐field conditions, biostimulants mainly influenced fruit metabolic profiles rather than directly enhancing growth or yield. Their performance appears closely tied to environmental factors, reinforcing the need for realistic, context‐specific evaluations to guide their effective integration into sustainable cropping systems.

## 1 Introduction

Southern Italy is increasingly subjected to variable and unpredictable environmental conditions, with water scarcity, rising temperatures, and erratic precipitation threatening natural resources and agricultural productivity (Cammarano et al. 2022). Among the most affected crops is tomato ( *Solanum lycopersicum* L.), a cornerstone of the region's economy and its cultural heritage. It is celebrated as one of the “protective foods” thanks to its impressive nutritional profile. Packed with lycopene, beta‐carotene, organic acids, flavonoids, and vitamin C, tomatoes play a vital role in promoting human health (Dorais et al. 2008). Indeed, numerous studies have highlighted a strong link between tomato consumption and a lower risk of certain cancers, cardiovascular diseases, and age‐related macular degeneration (Giovannucci 2002; Raiola et al. 2014). This protective effect is largely attributed to vitamins and carotenoids, which act as powerful antioxidants (Mayne 1996). By 2022, global tomato production had reached approximately 186.2 million Mg, with China contributing to 37% of the total output. In 2023, production increased further by 3%, reaching 191.8 million Mg, with 25% being destined for the production of canned products (FAOSTAT 2025; WPTC 2025). This trend reflects the rising market demand for export and domestic consumption, driven in part by the rapid population growth, which increased by 50% between 1961 and 2010 and continues to grow (FAOSTAT 2025).

Processing tomatoes, which are grown under open‐field conditions for industrial transformation purposes, demand high volumes of irrigation water, thus being particularly sensitive to year‐to‐year fluctuations in temperature and water availability that can severely affect both yield and fruit quality (Bisbis et al. 2018; Cammarano et al. 2022). Drought stress reduces plant fertility and accelerates fruit ripening, shortening the period for pigment accumulation and increasing oxidative stress, which results in uneven coloration (i.e., high incidence of sunscald fruits) and reduced transformation suitability (i.e., lower soluble solids content and higher pH values) (Bertin and Génard 2018; Bisbis et al. 2018). Reduced soil moisture can limit nutrient availability and uptake, while prolonged drought can degrade soil structure and reduce root access to essential resources (Bhattacharya 2021). The combination of premature senescence and altered metabolite composition may increase the fruit perishability and undermine the efficiency of industrial processing, raising operational expenses and affecting consumers' acceptance (Ripoll et al. 2014).

Addressing these challenges requires innovative agricultural solutions, including biostimulants, optimized irrigation systems, and other sustainable practices (e.g., the use of anti‐transpirants, grafting, biodegradable mulching, soil amendments with biochar, compost, superabsorbent polymers, etc.) (Khapte et al. 2019). Such approaches are essential to enhance crop resilience, maintain productivity, and support the livelihoods of farming communities, which are dependent on tomato cultivation (Bindi and Olesen 2011). These measures are critical for adapting to current climatic conditions and ensuring the long‐term sustainability of tomato production in a changing environment. One promising approach is the use of biostimulants, particularly plant‐based protein hydrolysates (PHs), which have shown potential to improve plant resilience and enhance yield and quality performance under drought‐stress conditions (Leporino et al. 2024; Patanè et al. 2025). These biostimulants contain bioactive compounds such as amino acids, peptides, and polysaccharides, which have been shown to significantly improve nutrient uptake, stimulate plant metabolism, and enhance resilience to abiotic stress (Colla et al. 2015; Wise et al. 2024). Their mode of action involves regulating key metabolic pathways that are critical during periods of stress. For instance, amino acids and peptides can activate enzymatic processes essential for carbon and nitrogen metabolism, ensuring an efficient redistribution of nutrients and osmolytes to maintain growth under adverse conditions. Some peptides are also linked to improved water use efficiency and oxidative stress management by modulating the production of reactive oxygen species and supporting cellular detoxification mechanisms. Under reduced photosynthetic efficiency, these bioactive compounds play a pivotal role in activating alternative carbon metabolism pathways, stabilizing cellular functions, and promoting adaptive signaling processes that sustain plant health and productivity (Colla, Cardarelli, et al. 2017). By modulating key metabolic pathways, biostimulants may help plants cope with environmental constraints such as heat and drought stress (Leporino et al. 2024; Carillo et al. 2025). Recent literature has also highlighted the importance of evaluating their effects under variable field conditions and tailoring their application to specific environmental contexts (Carillo 2025).

Despite the potentially beneficial effects, plant‐based PHs, edaphic factors, and genotype can strongly influence their effectiveness on yield and quality, highlighting the need for tailored applications based on specific soil conditions and cultivar characteristics (Caruso et al. 2019; Abidi et al. 2024; Golin et al. 2024). Indeed, although the interest in their use is growing, the actual impact of biostimulants under open‐field conditions remains difficult to predict. Thus, this study aimed to evaluate whether two commercial plant‐based biostimulants (MU, a formulation based on seaweed and plant‐derived amino acids, and SR, a potassium‐rich botanical extract enriched with amino acids and polysaccharides) could improve the fruit quality of processing tomatoes under field conditions. Their effects on yield performance and technological, nutritional, and functional traits of the “H1534” processing tomato cultivar were assessed over a two‐year open‐field trial in southern Italy (Foggia). We hypothesized that their efficacy would be strongly influenced by environmental variability and that they would act more as metabolic modulators than as direct promoters of yield, particularly under suboptimal conditions. Previous studies have reported beneficial effects of SR on fruit quality in crops such as orange (Massenti et al. 2012) and grapevine (Ziosi et al. 2012), mainly by accelerating and synchronizing ripening under different irrigation regimes. By contrast, no published data are currently available on the effects of MU on fruiting vegetable crops. Therefore, this work also aimed to verify whether these biostimulants, originally formulated to enhance technological fruit quality, could offer consistent benefits across seasons. Given the frequent exposure of processing tomatoes to variable climatic and biotic pressures, which often compromise yield and processing quality, their integration into sustainable farming systems must be carefully evaluated under operating conditions. Indeed, their effectiveness is not guaranteed and may vary significantly depending on seasonal environmental and agronomic contexts (Carillo 2025).

## 2 Materials and Methods

### 2.1 Experimental Site

An open‐field trial was conducted on a farm located in southern Italy (Foggia, Apulia Region, 41°32′48.8″N 15°36′11.6″E, 39 m a.s.l.) across two growing seasons (2019–2020). Crop rotation was performed with cauliflower and broad bean. The soil was classified as Haploxerepts according to USDA classification (Baillie 2001) with the following characteristics: 41% sand, 20% silt, 39% clay, 19.3 g kg^−1^ limestone, pH 7.52, 22.9 g kg^−1^ organic matter, 1.19 mS cm^−1^ electrical conductivity, 1.66‰ total N, 10.6 mg kg^−1^ available P, 218 mg kg^−1^ exchangeable K. The climate of Foggia is semi‐arid (Francaviglia and Di Bene 2019), and the annual sum of rainfall is 427 mm, on average, although almost absent during the summer period (Toreti et al. 2013).

### 2.2 Experimental Design and Crop Management

In both years, “H1534” processing tomato hybrid (Furia Seed, Monticelli Terme, PR, Italy) was transplanted at a four‐leaf stage in paired rows spaced 1.8 m (0.4 m between rows in pair), with a planting density of 2.78 plants m^−2^. Transplantations were performed on 16 April 2019 and 19 May 2020. A randomized block design with three replications was adopted, and each experimental plot had three 4‐m‐long paired rows. Irrigation, fertilization, pest control, and weed management were performed according to the cultivation guidelines of the Apulia Region (Italy) (APO Foggia 2020). The irrigation management replenished 100% crop evapotranspiration (Allan et al. 1998) and supplied 464 mm in 2019 (22 irrigation events) and 429 mm in 2020 (19 irrigation events). The irrigation turns, calculated as cycle duration divided by the number of irrigations, were 4 days (2019) and 5 days (2020), while the depth, derived by dividing the seasonal irrigation volume by the number of irrigation events, was 21 and 23 mm, respectively. The irrigation events were triggered once readily available water, monitored by capacitive probes (10 HS sensors, Meter Group Inc.), was scarce, and water volumes were estimated by multiplying reference evapotranspiration (Penman–Monteith method) by Kc, as reported by Burato et al. (2024). The drip‐irrigation system was made of a single plastic drip line lying in the middle of each paired row, and irrigation water followed FAO guidelines for agricultural purposes. Fertilization was applied throughout the growing season according to crop phenology, with application rates and timing adjusted based on projected fruit yield, soil characteristics, and the crop's nutritional demand. In 2019, 181 kg N ha^−1^, 121 kg P_2_O_5_ ha^−1^, and 99 kg K_2_O ha^−1^ were applied throughout the crop cycle, while 135 kg N ha^−1^, 110 kg P_2_O_5_ ha^−1^, and 68 kg K_2_O ha^−1^ were supplied to the crop in 2020. Overall, approximately 20% of N, 60% of P, and 20% of K were applied before transplant (as diammonium sulfate and complex fertilizers), while the remaining amounts were supplied to the crop through fertigation. In both years, weed control was performed through three interventions: (1) before transplanting (Pendimethalin 38.9%, at a dose of 1.75 L ha^−1^); (2) 15 days after transplanting (Metribuzin 52.17%, at a dose of 0.45 L ha^−1^); (3) during fruit development, using mechanical control. Crop protection was adopted against the following phytophagous insects: *Aphis* spp., *Tuta absoluta* (Meyrick), *Frankliniella occidentalis* (Pergande), *Heliothis armigera* (Hübner), *Spodoptera littoralis* (Boisduval), and *Tetranychus urticae* (Koch). Commercial insecticides containing Acetamiprid, Spinosad, and Spiromesifen as active ingredients were applied through foliar application. Two commercial liquid biostimulant products, namely MU (MATUR UP Fertenia Srl) and SR (SUNRED Biolchim S.p.A), were tested and compared with untreated control (CTRL). MU contains seaweed and plant extracts ( *Yucca schidigera* ), enriched with amino acids (methionine, arginine and phenylalanine). It contains 1.5 g L^−1^ of total N, 4.6 g L^−1^ of P, 3.5 g L^−1^ of K, with an electrical conductivity (EC) 0.51 dS cm^−1^, pH 6.0, and a specific weight of 1.30 kg L^−1^. It also includes 38.2 g L^−1^ of CaO, 39.7 g L^−1^ of MgO, 3.8 g L^−1^ of B, and 2.3 g L^−1^ of Fe chelated with EDTA. SR is a botanical extract‐based biostimulant containing oxylipins, amino acids (methionine, phenylalanine), monosaccharides, and derivatives from inorganic fertilizers (potassium salts, urea). The formulation includes 26.6 g L^−1^ of organic N, 13.3 g L^−1^ of mineral N, 93.1 g L^−1^ of K, and 186.2 g L^−1^ of organic C, with a pH of 8.1, and a specific weight of 1.33 kg L^−1^ (Massenti et al. 2012; Ziosi et al. 2012). MU and SR were uniformly sprayed twice on the canopy respectively at ~40 and ~30 days before the expected harvesting (on 15 and 25 July 2019, and on 27 July and 3 August 2020), starting from the breaking‐color stage of the first truss, according to manufacturers' recommendation. They were applied using a 25 L stainless steel sprayer, at a concentration of 3.5 mL L^−1^ H_2_O (MU) and 8.0 mL L^−1^ H_2_O (SR) with a solution volume of 1000 L ha^−1^. The untreated control was sprayed with tap water only. The amount of nutrients supplied through the treatments was negligible compared with the crop nutritional demand (on average, 0.21%, 0.02%, and 1.13% on the total amount of N, P_2_O_5_, and K_2_O per ha). Harvesting was performed by hand and took place on 20 August 2019 and on 2 September 2020. All plots were harvested simultaneously when marketable fruits nearly accounted for 85% of the total.

### 2.3 Weather Conditions

**FIGURE 1:**
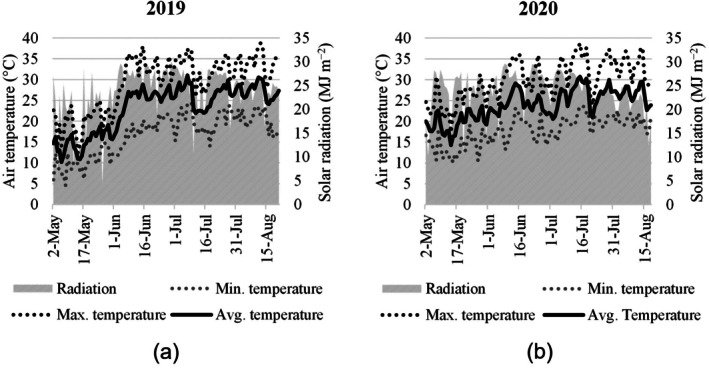
Weather conditions over two growing seasons (a: 2019, b: 2020), with the minimum (grey dashed line), average (black solid line), and maximum (black dashed line) air temperatures (°C), and solar radiation (area with diagonal stripes, MJ m^−2^) reported.

Weather data were collected from a local weather station (Figure 1). In 2019, the seasonal rainfall amounted to 102.0 mm, while it decreased to 89.0 mm in 2020. In 2019, the average maximum and minimum temperatures were 29.3°C and 16.9°C, respectively, with extreme values reaching 38.8°C and 4.5°C. The overall average temperature for the year was 23.1°C. In 2020, air temperatures were notably higher compared with 2019. The average maximum temperature increased to 30.1°C (maximum 38.7°C), while the average minimum temperature rose to 17.7°C (minimum 10.1°C). The overall average temperature for the year was 23.9°C.

### 2.4 Fruit Yield and Defects

At harvesting, yield performance was assessed on 10 representative plants on the central twin row of each plot. Marketable and total yield, green, and rotten fruits, the number of marketable fruits *per* plant, and the average fruit weight were assessed as reported in Burato et al. (2024). The incidence of defective fruits (%), mainly referred to as viral symptoms, was determined on a random pool of 100 marketable fruits per plot (Fusco et al. 2023).

### 2.5 Fruit Analyses

To evaluate fruit technological traits and the profiles of primary and secondary metabolites, mature, asymptomatic fruits were collected from different plants subjected to the same treatment within each plot. For each biological replicate, 10 fruits from different plants, ~750–800 g of fruit tissue, were washed, dried, and homogenized. Fresh homogenates were either immediately analyzed or stored at −80°C until further analyses.

#### 2.5.1 Technological Traits

Fruit titratable acidity (expressed as grams of citric acid *per* 100 g of FW juice, or g% CA), pH, soluble solids content (°Brix), and dry matter content (expressed as g of dry matter *per* 100 g of FW juice, of g%) were determined as reported in Burato et al. (2025). Based on these parameters, Brix yield (marketable yield × soluble solids content/100) and soluble solids content‐to‐titratable acidity ratio were then calculated according to Parisi et al. (2022).

#### 2.5.2 Starch and Soluble Carbohydrates Content

Soluble sugars were quantified in ethanolic extracts of fresh tomato fruits according to Fusco et al. (2023). Samples were sequentially extracted with ethanol–water solutions, and the pooled supernatants were used to determine glucose, fructose, and sucrose content via an enzymatic assay coupled with pyridine nucleotide reduction. Absorbance at 340 nm was measured using a Synergy HT spectrophotometer (BioTEK Instruments), and results were expressed as mg g^−1^ FW. Starch in the residual pellet was hydrolyzed by alkaline and enzymatic treatment (α‐amylase and amyloglucosidase), and the resulting glucose was quantified using the same method. The fructose‐to‐glucose ratio and total sweetness index (TSI) were then calculated, with TSI determined according to Baldwin et al. (1998).

#### 2.5.3 Polyphenols and Lycopene Content

Polyphenol content (μg g^−1^ FW) was determined using the Folin–Ciocalteu method according to Singleton et al. (1999), with slight modifications reported by Burato et al. (2024). Extracts from fresh tomato fruits were reacted with diluted Folin–Ciocalteu reagent and sodium carbonate, and absorbance at 760 nm was measured using a Synergy HT microplate reader (BioTEK Instruments). Results were expressed as gallic acid equivalents (GAE). Lycopene content (mg g^−1^ FW) was assessed following the protocol by Sadler et al. (1990). Fresh samples were extracted with a hexane:acetone:methanol mixture (2:1:1, v:v), containing 0.05% BHT to prevent oxidation. After centrifugation, the absorbance of the hexane phase was read at 472 nm and quantified using a standard curve of pure lycopene.

#### 2.5.4 Proteins and Free Amino Acids Content

Soluble proteins were extracted from fresh tomato fruits using a Tris–HCl/MgCl₂ buffer and quantified by the Bradford assay (Bradford 1976), using bovine serum albumin (BSA) as the standard. Absorbance at 595 nm was measured with a Synergy HT microplate reader (BioTEK Instruments), and protein content was expressed in mg g^−1^ FW. Free amino acids were extracted in 60% ethanol and quantified by high‐performance liquid chromatography (HPLC) after precolumn derivatization with *o*‐phthaldialdehyde (OPA), following Carillo et al. (2019). In the same extract, proline content was determined by a colorimetric assay based on the ninhydrin method of Bates et al. (1973), with modifications from Carillo et al. (2019). Amino acids were expressed as μmol g^−1^ FW.

### 2.6 Statistical Analysis

Three independent biological replicates per treatment were used. Each biological replicate (~750–800 g) consisted of 10 fully ripe fruits, each harvested from a different plant within the same treatment plot. Data were analyzed by ANOVA using the SPSS 25 software package, after verifying the assumptions of normality and homogeneity of variance using the Shapiro–Wilk and Levene's tests, respectively. All variables fulfilled the required assumptions. Means were compared by Tukey's HSD test at *p* < 0.05. The heat map was generated using the https://biit.cs.ut.ee/clustvis/ (accessed February 20, 2025) online program package with Euclidean distance as the similarity measure and hierarchical clustering with complete linkage. The pathway map was calculated as Logarithm base 2 (Log_2_) of the value to average (avg.) ratio and visualized using a false‐color scale, with red indicating an increase and blue a decrease of values.

## 3 Results

### 3.1 Agronomic Performance

The treatment factor (T) did not affect the agronomic performance of processing tomatoes cv. “H1534.” Total yield, marketable yield, average fruit weight, and the number of marketable fruits per plant showed no significant differences among treatments (Table 1). Conversely, the year (Y) had a strong effect on all major yield components. In 2020, total and marketable yield decreased by 45% and 44%, respectively, when compared with 2019, with a severe increase in the incidence of defective fruits (37% in 2020 and 9% in 2019). The number of marketable fruits per plant was also lower in 2020 (42.9) compared with 2019 (78.7), while the average fruit weight (67.3 g, on average) remained unaffected by Y. On average, over the two‐year period, marketable and total yield were, respectively, 113.1 and 140.3 Mg ha^−1^ (81% of marketable fruits on total) (Table 1). A significant T × Y interaction was observed only for the incidence of defective fruits. In 2020, the SR treatment showed the highest proportion of defects (46%), which was 11% higher than MU and 16% higher than the control in the same year (Table 1).

**TABLE 1:** Effects of the commercial biostimulants MU and SR compared with control (CTRL) on yield and its components, fruit weight, plant fertility, and fruit defects recorded on processing tomatoes cv. “H1534.”

	Unripe fruits (Mg ha^−1^)	Rotten fruits (Mg ha^−1^)	Marketable yield (Mg ha^−1^)	Total yield (Mg ha^−1^)	Average fruit weight (g)	No. of ripe fruits/plant	Total defective fruits (%)
*Treatment (T)*							
CTRL	4.77 ± 1.46	2.96 ± 1.04	112.4 ± 7.05	137.86 ± 6.41	66.82 ± 2.10	61.07 ± 2.37	21.00 ± 0.02
MU	5.72 ± 1.93	4.35 ± 1.72	107.1 ± 4.68	140.61 ± 2.16	66.14 ± 2.20	58.55 ± 3.08	22.00 ± 0.03
SR	4.31 ± 1.12	3.02 ± 0.58	119.8 ± 7.54	142.45 ± 7.66	69.00 ± 1.87	62.74 ± 4.62	27.00 ± 0.01
*Year (Y)*							
2019	4.90 ± 1.42	3.64 ± 1.48	144.8 ± 5.40 a	180.54 ± 3.52 a	66.35 ± 2.38	78.70 ± 2.62 a	9.00 ± 0.02 b
2020	4.96 ± 1.59	3.25 ± 0.74	81.42 ± 7.45 b	100.07 ± 7.30 b	68.29 ± 1.73	42.87 ± 3.79 b	37.00 ± 0.02 a
*T × Y*							
CTRL × 2019	4.03 ± 0.95	2.64 ± 1.50	143.9 ± 5.35	177.44 ± 8.87	64.98 ± 2.83	79.77 ± 0.54	12.00 ± 0.02 a
MU × 2019	5.43 ± 2.41	4.38 ± 1.93	139.2 ± 5.57	180.94 ± 0.84	65.57 ± 1.53	76.49 ± 3.49	9.00 ± 0.02 a
SR × 2019	5.24 ± 0.90	3.89 ± 1.02	151.4 ± 5.28	183.24 ± 0.84	68.50 ± 2.78	79.86 ± 4.74	8.00 ± 0.02 a
CTRL × 2020	5.50 ± 1.97	3.28 ± 0.59	80.96 ± 8.76	98.27 ± 3.94	68.65 ± 1.37	42.38 ± 4.21	30.00 ± 0.01 c
MU × 2020	6.00 ± 1.45	4.32 ± 1.51	75.01 ± 3.79	100.28 ± 3.49	66.71 ± 2.87	40.62 ± 2.67	35.00 ± 0.04 c
SR × 2020	3.38 ± 1.35	2.15 ± 0.13	88.28 ± 9.80	101.67 ± 14.48	69.51 ± 0.96	45.62 ± 4.50	46.00 ± 0.01 b
*Significance*							
T	ns	ns	ns	ns	ns	ns	ns
Y	ns	ns	**	**	ns	***	***
T × Y	ns	ns	ns	ns	ns	ns	*

*Note:* All data are presented as mean ± standard error, *n* = 3. Different letters indicate significant mean differences according to Tukey's HSD test (*p* < 0.05). *, **, and *** denote significant effects at *p* ≤ 0.05, *p* ≤ 0.01, and *p* ≤ 0.001, respectively.

Similar to what was reported for yield traits, T did not affect technological quality (Table 2). In contrast, the Y effect had a significant impact on all evaluated traits. In 2020, soluble solids content, dry matter content, and titratable acidity increased by 5%, 7%, and 20%, respectively, compared with 2019. Conversely, pH, soluble solids content‐to‐titratable acidity ratio, and Brix yield decreased by 7%, 13%, and 41%, respectively, compared with the first year of the trial. A significant T × Y interaction was observed for most of the fruit technological traits (Table 2). Indeed, dry matter content increased in all treatments in 2020 with respect to CTRL × 2019, while pH decreased in CTRL in 2020 in comparison with 2019 (−8%) and was lower in MU × 2020 than MU × 2019 (−11%). Conversely, SR reached comparable values of pH and titratable acidity over the 2 years. As regards titratable acidity, both CTRL and MU recorded higher values in 2020 than in 2019. The soluble solids content‐to‐titratable acidity ratio was lower in MU × 2020 compared with MU × 2019 (−18%).

**TABLE 2:** Effects of the commercial biostimulants MU and SR compared with control (CTRL) on fruit technological traits and Brix yield in processing tomato cv. “H1534.”

Treatment	Soluble solids content (°Brix)	Dry matter (g%)	Titratable acidity (g% CA)	pH	Soluble solids content/titratable acidity	Brix yield (Mg ha^−1^)
*Treatment (T)*						
CTRL	5.50 ± 0.06	6.24 ± 0.12	0.48 ± 0.01	4.42 ± 0.03	11.54 ± 0.11	6.17 ± 0.43
MU	5.38 ± 0.16	6.46 ± 0.15	0.46 ± 0.01	4.56 ± 0.09	11.88 ± 0.22	5.72 ± 0.40
SR	5.41 ± 0.11	6.32 ± 0.12	0.46 ± 0.02	4.51 ± 0.06	11.78 ± 0.25	6.42 ± 0.36
*Year (Y)*						
2019	5.29 ± 0.10 b	6.12 ± 0.13 b	0.42 ± 0.01 b	4.65 ± 0.08 a	12.52 ± 0.25 a	7.66 ± 0.31 a
2020	5.57 ± 0.11 a	6.55 ± 0.13 a	0.51 ± 0.01 a	4.34 ± 0.04 b	10.95 ± 0.14 b	4.54 ± 0.48 b
*T × Y*						
CTRL × 2019	5.43 ± 0.04	5.96 ± 0.11 b	0.45 ± 0.01 bc	4.60 ± 0.04 ab	12.16 ± 0.03 ab	7.82 ± 0.35 a
MU × 2019	5.22 ± 0.14	6.38 ± 0.13 ab	0.40 ± 0.01 b	4.82 ± 0.16 a	13.05 ± 0.36 a	7.28 ± 0.47 ab
SR × 2019	5.22 ± 0.12	6.03 ± 0.14 ab	0.42 ± 0.02 b	4.53 ± 0.04 ac	12.35 ± 0.35 ab	7.88 ± 0.11 a
CTRL × 2020	5.57 ± 0.07	6.52 ± 0.13 a	0.51 ± 0.01 a	4.24 ± 0.02 c	10.92 ± 0.19 ab	4.51 ± 0.50 c
MU × 2020	5.53 ± 0.18	6.53 ± 0.18 a	0.52 ± 0.02 a	4.30 ± 0.03 bc	10.71 ± 0.08 b	4.16 ± 0.33 c
SR × 2020	5.60 ± 0.10	6.61 ± 0.09 a	0.50 ± 0.02 ac	4.48 ± 0.08 ac	11.21 ± 0.15 ab	4.96 ± 0.61 bc
*Significance*						
T	ns	ns	ns	ns	ns	ns
Y	*	*	**	***	**	***
T × Y	ns	*	***	*	*	***

*Note:* All data are presented as mean ± standard error, *n* = 3. Different letters indicate significant mean differences according to Tukey's HSD test (*p* < 0.05). *, **, and *** denote significant effects at *p* ≤ 0.05, *p* ≤ 0.01, and *p* ≤ 0.001, respectively.

### 3.2 Soluble Sugars, Starch, and Secondary Metabolites

**FIGURE 2:**
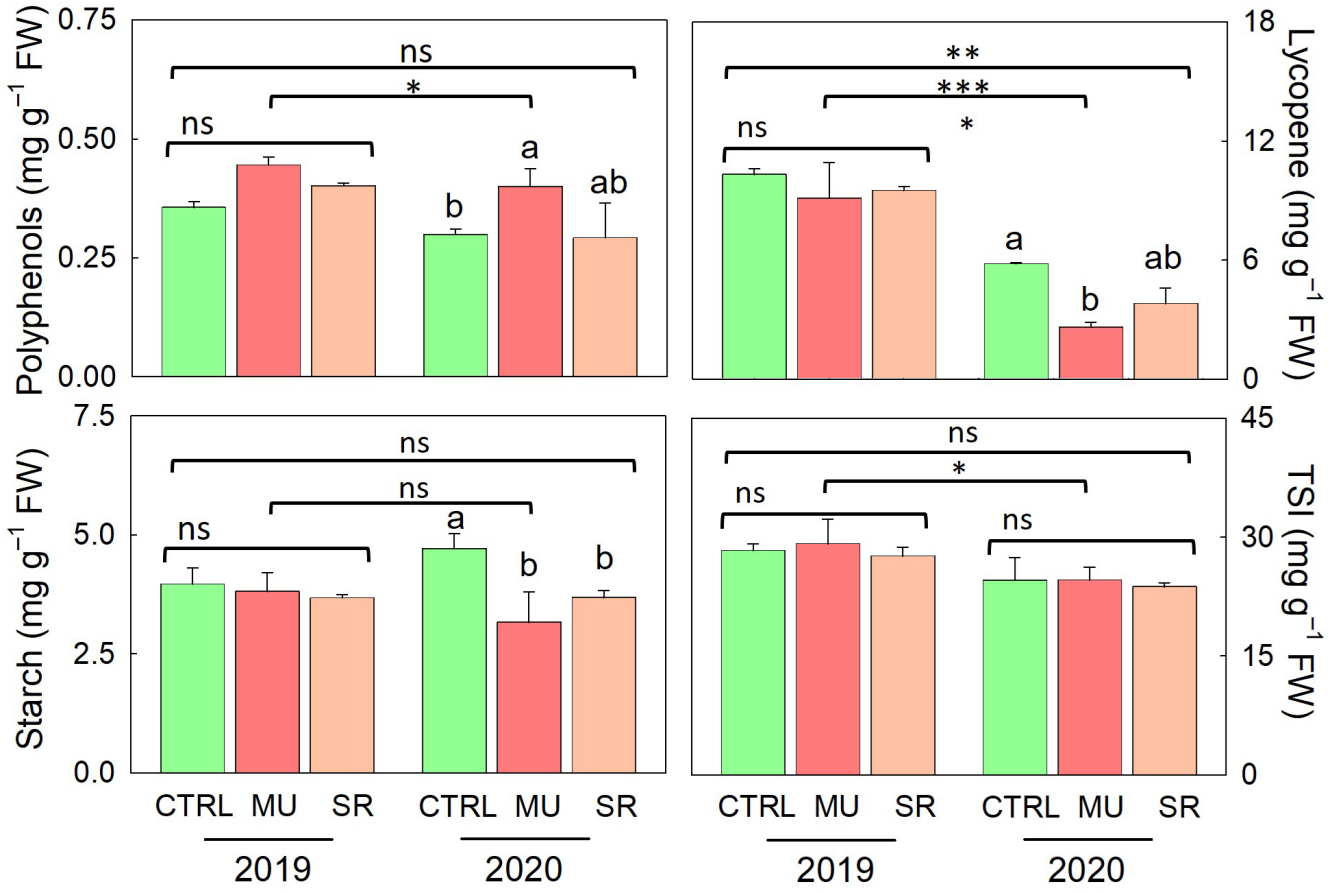
Effects of the commercial biostimulants MU and SR compared with control (CTRL) on polyphenols, lycopene, total sweetness index (TSI), and starch, in processing tomato cv. “H1534.” All data are presented as mean ± standard error, *n* = 3. Different letters indicate significant mean differences according to Tukey's HSD test (*p* < 0.05). *, **, and *** denote significant effects at *p* ≤ 0.05, *p* ≤ 0.01 and *p* ≤ 0.001, respectively.

The analysis revealed limited T effects on the measured metabolites. Among them, MU significantly increased polyphenol content by 27% compared with the control (Figure 2). No significant difference was observed between MU and SR. Similarly, no significant treatment effects were found for lycopene, total sweetness index (TSI), fructose‐to‐glucose ratio, or starch content (Figure 2; Table S1). The Y factor had a significant influence on several key metabolites. In 2019, TSI, polyphenol, and lycopene levels were significantly higher than in 2020, with increases of 18%, 21%, and 137%, respectively (Figure 2). In contrast, starch content was not significantly affected by the year factor. A significant T × Y interaction was observed for lycopene. In 2020, lycopene concentration in MU‐treated plants was markedly reduced by 55% compared with the control, while the difference between MU and SR was not statistically significant (Figure 2).

### 3.3 Proteins and Free Amino Acids

**FIGURE 3:**
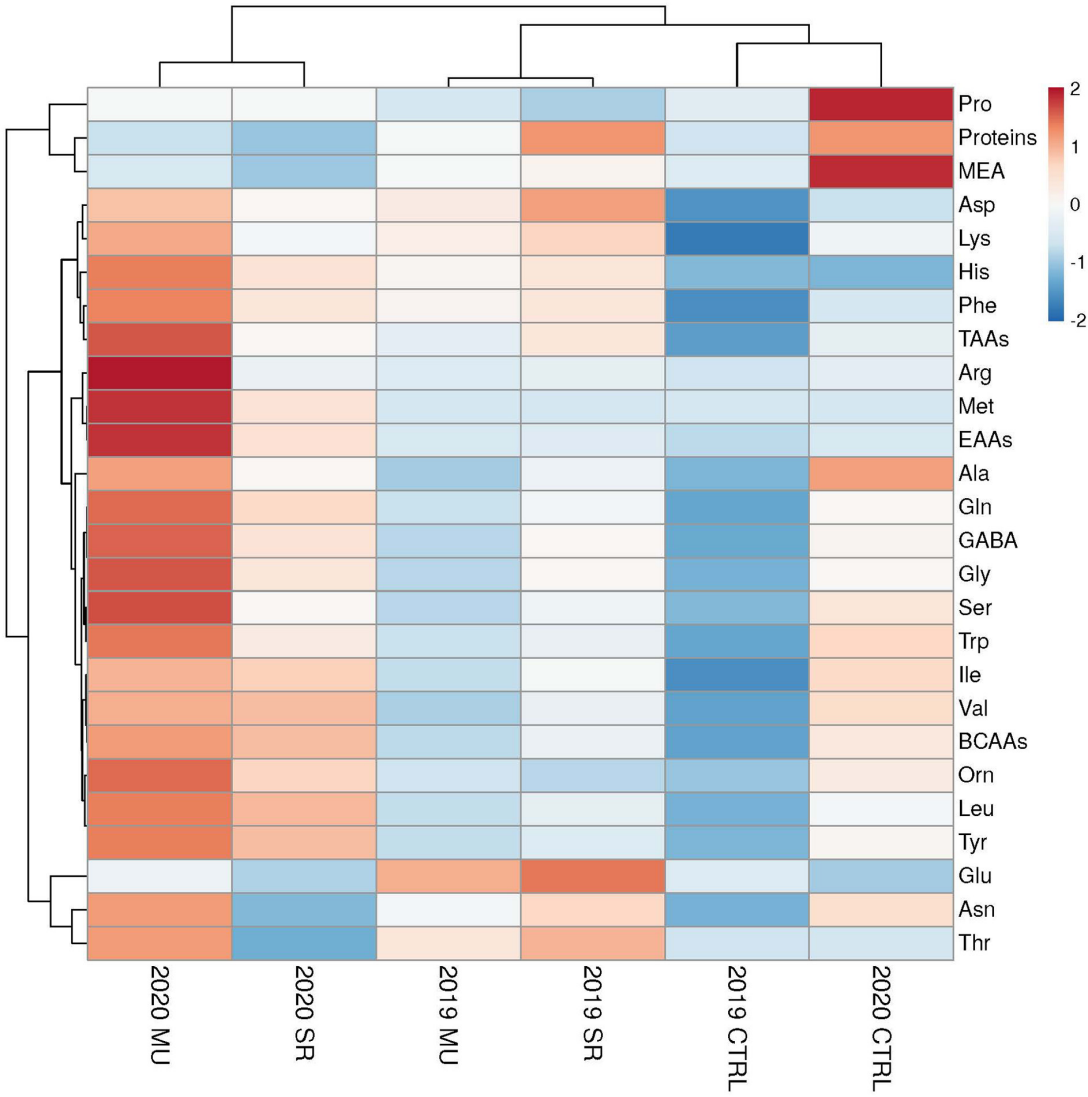
Cluster heat map analysis summarizing the effects of the commercial biostimulants MU and SR compared with control (CTRL) on soluble proteins and free amino acids in processing tomato cv. “H1534.” Ala, alanine; Arg, arginine; Asn, asparagine; Asp, aspartate; BCAAs, branched chain amino acids; EAAs, essential amino acids; GABA, γ‐aminobutyric acid; Gln, glutamine; Glu, glutamate; Gly, glycine; His, histidine; Ile, isoleucine; Leu, leucine; Lys, lysine; MEA, monoethanolamine; Met, methionine; Or, Ornithine; Phe, phenylalanine; Pro, proline; Ser, serine; TAAs, total amino acids; Thr, threonine; Tyr, tyrosine; Trp, tryptophane; Val, valine.

The heatmap (Figure 3) clearly represents how soluble proteins and amino acids were influenced by the T × Y interaction. Regarding T effects, in 2019, SR significantly increased soluble protein content by 40% compared with the control (Table S2). However, no treatment effects were observed for total amino acids (TAAs), branched‐chain amino acids (BCAAs), or essential amino acids (EAAs), when considered independently of year. In contrast, the Y had a strong effect on amino acid profiles. In 2020, BCAAs and EAAs increased significantly, by 51% and 125%, respectively, compared with 2019 (Table S2). Among individual amino acids, substantial increasing trends were observed for arginine, isoleucine, leucine, tryptophan, and valine, which rose between 39% and 121% (red tones within the 2020 columns in Figure 3; Table S2). Other amino acids displayed similar year‐dependent shifts, with alanine, γ‐aminobutyric acid (GABA), glutamine, glycine, ornithine, proline, serine, and tyrosine increasing by 43%–85% in 2020. Contrary to this, glutamate decreased by 27% (Table S2). These trends are visually represented by red intensities in the 2020 columns of Figure 3. The heatmap (Figure 3) shows an apparent clustering by Y, with 2020 samples distinct from those of 2019 and by T, with MU‐ and SR‐treated fruits forming distinct groups within each year. These patterns reflect specific changes in amino acid profiles driven by both environmental conditions and biostimulant application. In 2020, MU significantly increased arginine, histidine, leucine, and ornithine levels (by 32%–260% vs. the control), while both MU and SR reduced MEA and proline contents (by 32%–39%) (Table S2). These results confirm that MU and SR triggered distinct metabolic responses under stress.

## 4 Discussion

Understanding the effects of plant‐based biostimulants on processing tomato under operating conditions requires a careful evaluation of different sources of variation. In this section, we examine the results by first focusing on treatment effects (T), then on year‐related differences (Y), and finally on the interaction between treatments and year (T × Y), to better interpret the response of yield, fruit quality, and plant metabolism under variable environmental conditions.

The application of PHs (MU and SR) did not significantly affect the agronomic and qualitative technological traits assessed in our research, in contrast with previous studies performed on processing tomatoes under open‐field conditions. Indeed, Cozzolino et al. (2021), comparing the effect of three biostimulants (an extract of brown seaweed *Ecklonia maxima* (Osbeck), a legume‐derived protein hydrolysate, and a tropical plant extract), reported an increase of +18% in marketable yield as effect of PHs over untreated processing tomato cv. “Coronel.” Rouphael et al. (2021), using plant extract Auxym, found even higher yield gains on “San Marzano” landraces (+34%), while Caruso et al. (2019), assessing the same commercial product and “Trainer” biostimulant, observed more contained increases on “Piennolo del Vesuvio” tomato ecotype (+12%). Under greenhouse conditions, Colla, Hoagland, et al. (2017) showed that foliar applications of “Trainer” boosted total tomato yield by over 6.6% to the control. Contrasting results were found in literature about the foliar application of PHs on tomato crop (Cozzolino et al. 2021; Antonucci et al. 2023), suggesting to better investigate the interaction among biostimulants composition, phenological stage, distribution time, and application dose, and how these factors can affect the absorption of biostimulants through stomata and their use efficiency (Golin et al. 2024).

The lack of significant effects induced by T suggests that the number of applications, being only two in our trial, may have been insufficient to boost yield, as most cited studies applied PHs four times, despite four applications being rarely feasible in operating farming conditions due to costs and labor constraints. Indeed, it should be noted that, considering the current selling prices of PHs and average costs needed for spraying processing tomatoes (Burato et al. 2025), four MU and SR applications would approximately require EUR 300 ha^−1^ and EUR 800 ha^−1^, respectively. This, combined with scarce availability of qualified labor (license for agricultural tractors), especially in a heavy workload period such as pre‐harvesting, would be unfeasible for most tomato growers if not providing a marketable yield increase of at least 10 Mg ha^−1^, given a hypothetical selling price of EUR 150 Mg^−1^ (Burato et al. 2024). MU‐treated plants displayed moderate fruit production with a balanced proportion of marketable and defective fruits, which aligns with previous findings showing a biostimulant‐induced metabolic modulation rather than a direct yield enhancement (Rouphael et al. 2017). However, it is important to note that the PHs tested in this study, applied according to manufacturer‐recommended doses and timing, were primarily intended to enhance fruit quality rather than to increase yield performance. Subramaniyan et al. (2023) assessed three doses of a seaweed extract (Kendal Root), namely 2.5, 5.0, and 10 L ha^−1^, and found that only the highest dose resulted in heightened yield and plant growth. Additionally, biostimulant effects are genotype‐dependent and, thus, MU and SR could have better performances if tested on other cultivars (Rouphael et al. 2021). In fact, different studies have highlighted that the effect of biostimulants on yield and its components, as well as on nutritional and functional fruit quality, can be strongly influenced by genotype and growing conditions. Vultaggio et al. (2025) evaluated the response of two tomato genotypes (“Creativo” F1 and “P1” ecotype) to the application of three biostimulants, highlighting that yield was more enhanced in the hybrid, while the ecotype showed better improvements in technological quality when all three products were combined. Similarly, Patanè et al. (2025) reported genotype‐specific responses in four tomato cultivars under two irrigation regimes, showing that PHs may improve or worsen technological quality depending on cultivar and water availability. Therefore, further research should explore the effect of MU and SR on other tomato varieties suitable for different canning purposes (e.g., peeled, cherry‐type), across multiple locations and under variable environmental conditions.

While the application of biostimulants did not significantly influence total yield, differences in fruit quality parameters were noted. The technological quality of tomato fruit, including soluble solids, dry matter, pH, and titratable acidity, is fundamental for its suitability for industrial processing. MU‐treated fruits showed a marginally higher pH and dry matter content than the control and SR, yet these differences were not statistically significant. The pH result aligned with previous studies, indicating that plant extract‐based biostimulants tend to have minimal effects on fruit acidity (Povero et al. 2016; Patanè et al. 2025). Conversely, the trend observed in dry matter content is more complex and warrants further consideration. While MU‐treated fruits exhibited a slightly higher dry matter percentage, this did not translate into a significant improvement, raising questions about its influence on fruit composition. In previous studies, plant‐derived biostimulants have been linked to increased dry matter, often associated with improved firmness and post‐harvest resilience (Cozzolino et al. 2021; Burato et al. 2024). However, in this case, the lack of a clear effect may indicate that the biostimulant mode of action does not necessarily promote structural reinforcement of the fruit.

In terms of biochemical responses, MU‐treated plants exhibited higher concentrations of arginine, histidine, leucine, and ornithine, indicating a potential role in nitrogen metabolism. Conversely, MU reduced proline, a well‐known stress marker (Szabados and Savouré 2010), suggesting that it may have modulated the plant's physiological adaptation to stress.

The Y had a substantial impact on determining the number of ripe fruits per plant, marketable and total yield, and incidence of fruit defects. A significant reduction in fruit production was observed in the second year of the study, due to *Fusarium* spp. (tracheomycotic disease) and Tomato Spotted Wilt Orthotospovirus (TSWV) diseases, which have been reported to compromise fruit set and overall plant productivity (Fusco et al. 2023). Targeted treatments were applied in 2020 to control *Fusarium* and *Frankliniella occidentalis* (the insect vector of TSWV), partially limiting the spread of these pathogens. Nevertheless, reductions in plant development and fruit set were observed, aligned with the findings by Burato et al. (2024), who reported a decrease in vegetative and fruit dry biomass. This resulted in a critical reduction in Brix yield, considered a key parameter for assessing the profitability of processing tomatoes (Navez et al. 1999).

The Y factor also had a significant effect on key fruit quality technological attributes, according to extensive literature (Parisi et al. 2006; Ronga et al. 2017; Bertin and Génard 2018). The increase in titratable acidity and dry matter in the second year suggests an adaptive response to environmental stress, as plants tend to accumulate solutes under low water availability (Beckles 2012). This phenomenon has been widely observed in fruit crops subjected to suboptimal environmental conditions (McCormick et al. 2008). Consequently, the soluble solids content‐to‐titratable acidity ratio was significantly higher in 2019 than in 2020, reinforcing the notion that environmental stressors significantly influenced fruit metabolism. Indeed, when growing under suboptimal conditions, plants often modify their metabolic pathways to balance osmotic adjustments and energy distribution, possibly leading to shifts in sugar and organic acid accumulation (Ma et al. 2022).

In 2019, plants accumulated higher levels of both primary and secondary metabolites, reflecting more favorable growing conditions that promoted sugar biosynthesis and storage, as confirmed by the significantly higher TSI. Previous studies suggest that a higher sugar content supports improved photosynthetic efficiency and reduces photorespiration, ultimately contributing to better fruit quality (Ke et al. 2019). In contrast, 2020 was characterized by stress‐induced metabolic shifts, such as the suppression of carotenoid biosynthesis and protein accumulation, and a stimulation of antioxidant pathways like polyphenol synthesis. The reduction in sweetness index observed in 2020 may reflect altered sugar metabolism and source‐to‐sink dynamics under drought conditions, likely due to restricted phloem loading or impaired carbohydrate translocation. Such shifts can impact fruit taste and processing quality, especially when fructose content is reduced relative to glucose.

The interaction between T and Y emphasized that environmental constraints played a more dominant role than biostimulant application in determining fruit set and quality. The significant reduction in fruit production and the increased percentage of defective fruits in 2020 suggest that external stressors likely limited the effectiveness of the treatments. These findings reinforce the understanding that biostimulants alone may not fully mitigate the effects of environmental stressors, but can contribute to enhancing specific physiological traits of the plant (Povero et al. 2016).

In 2020, the SR treatment was associated with the highest marketable‐to‐total yield ratio (86.8%); although differences were not statistically significant, as well as the highest incidence of defective fruits. This suggests that SR may have influenced physiological processes related to fruit development, potentially increasing fruit load and accelerating ripening (Chrysargyris et al. 2020). On the other hand, MU showed a more balanced distribution between marketable and defective fruits, without a clear advantage in terms of yield performance, which further supports its role as a modulator of physiological responses rather than a direct enhancer of productivity.

Notably, MU significantly increased polyphenol content in 2020, whereas lycopene levels remained low across all treatments. This divergence suggests that, under stress, metabolic fluxes may have been redirected toward the phenylpropanoid pathway, which is more immediately responsive to oxidative signals (Rao and Zheng 2025). Phenylpropanoids, such as flavonoids, are synthesized earlier and respond more rapidly to oxidative stress signals than carotenoids, whose accumulation depends on sustained photosynthetic activity and plastid development (Agati et al. 2012). Recent findings confirm that metabolic competition exists between these two pathways, and the expression level of *SlPSY1* ( *Solanum lycopersicum* *Phytoene Synthase 1*) can modulate flux toward either carotenoid or flavonoid biosynthesis, ultimately affecting not only plant photosynthesis but also tomato fruit quality (Cao et al. 2024). The observed pattern may therefore reflect a stress‐induced shift in antioxidant strategy, with carbon rerouted from pigment and starch biosynthesis toward the production of rapidly activated antioxidant secondary metabolites. Consistently, starch levels in MU‐ and SR‐treated plants were lower in 2020 compared with the control, suggesting that the increased polyphenol accumulation may have occurred at the expense of carbohydrate storage.

In contrast, plants treated with SR, a formulation rich in oxylipins, maintained more stable protein content and exhibited a modest decrease in proline. These effects may be associated with the regulatory role of these compounds in stress responses and fruit ripening (Huguet‐Robert et al. 2003).

Burato et al. (2024) also identified a strong correlation between amino acid profiles and environmental stress responses, highlighting the role of biostimulants in modulating these metabolic pathways. MU‐ and SR‐treated fruits from 2020 form distinct clusters, primarily driven by increases in GABA, glutamate, arginine, and leucine, amino acids closely linked to drought‐related signaling, osmotic adjustment, and nitrogen remobilization (Carillo 2018; Wang et al. 2021; Heinemann and Hildebrandt 2021). These distinct clustering patterns suggest that MU and SR modulate stress‐response pathways through different mechanisms, likely reflecting their specific compositions. MU, containing calcium, magnesium, and seaweed extracts, may enhance nitrogen assimilation and stimulate phenolic metabolism, whereas SR, enriched in oxylipins and potassium, could primarily influence signaling and osmoprotective functions. Accordingly, the observed increase in GABA, arginine, and branched‐chain amino acids (BCAAs) under both treatments, particularly in 2020, points to a multifaceted physiological response to combined drought and oxidative stress. These metabolites are known to accumulate in fruit tissues as part of adaptive strategies supporting osmotic regulation, nitrogen storage, and redox balance (Kinnersley and Turano 2000; Obata and Fernie 2012). Arginine may also serve as a nitrogen reservoir or a precursor to polyamines under stress (Flores and Galston 1982). Nonetheless, such changes could partly reflect intrinsic developmental processes, such as fruit ripening or protein turnover, which can modulate amino acid levels independently of external inputs (Carrari and Fernie 2006). This suggests that biostimulant‐induced effects may overlap with, rather than replace, endogenous maturation pathways. The results of this study reflect a reality that researchers often encounter but rarely communicate under field conditions, especially under complex stress scenarios: biostimulants do not always deliver the expected agronomic outcomes (du Jardin et al. 2025). This should not be seen as a failure, but a reminder of the intrinsic complexity and variability of biological systems. Yet, there is a tendency, in both research and commercial contexts, to publish only positive outcomes. At the same time, neutral or inconclusive results are often left aside or retested to obtain clearer effects. From a practical perspective, farmers are generally cautious about adopting inputs that do not offer a clear yield return, especially under open‐field conditions where net incomes are often tight. While our findings reveal interesting metabolic changes, their practical relevance remains limited in the absence of effective agronomic or economic benefits, as long as no surplus values are yet recognized for nutraceutical properties on canned products. This gap between experimental interest and field adoption should be considered in future evaluations of biostimulants. Our results suggest that, under suboptimal and uncontrolled field conditions, plant‐based biostimulants may not consistently promote growth or yield but can still modulate key metabolic traits related to fruit quality. In particular, the enhancement of essential amino acids such as leucine, isoleucine, and phenylalanine under stress conditions may offer added nutritional value. Although yield gains were not observed, such compositional shifts could be relevant for high‐value processing chains or functional food applications. A realistic, context‐specific approach is essential to integrate biostimulants effectively into agricultural practice.

## 5 Conclusions

This study highlights the significant influence of environmental conditions on tomato yield, fruit quality, and metabolic responses. Although the biostimulants tested did not significantly improve yield traits and technological quality, they modulated specific metabolic pathways in processing tomato cv. “H1534” grown under southern Italian conditions. MU promoted polyphenol accumulation and altered nitrogen metabolism, while SR contributed to°Brix stability. However, the decline in lycopene, particularly with MU, suggests a metabolic trade‐off prioritizing stress responses over carotenoid biosynthesis.

The increase in essential and branched‐chain amino acids, especially under MU, indicates a metabolic shift toward stress adaptation rather than direct growth enhancement. A concurrent reduction in proline and protein content (in treated plants) further supports the hypothesis that biostimulants influenced stress perception and nitrogen allocation, rather than enhancing osmoprotection or protein synthesis. The control plants, by contrast, maintained stable protein levels.

From an economic perspective, the lack of yield improvement limits the immediate value of MU and SR for growers. However, the observed effects on fruit quality traits, such as amino acid profiles and °Brix, may offer potential in niche markets or processing contexts where nutritional traits are prioritized. By linking these findings to product composition and existing literature, this work contributes to a clearer understanding of how plant‐based biostimulants interact with crop metabolism under operating conditions. Future studies should refine these insights, aiming to develop crop‐ and environment‐specific strategies that improve biostimulant efficacy and consistency in sustainable agriculture.

## Author Contributions

**Giovanna Marta Fusco:** writing – review and editing, methodology, investigation, formal analysis, data curation, software, visualization. **Andrea Burato:** writing – review and editing, methodology, investigation, data curation, references. **Alfonso Pentangelo:** methodology, investigation. **Petronia Carillo:** writing – review and editing, writing – original draft, supervision, methodology, conceptualization, visualization. **Mario Parisi:** writing – review and editing, writing – original draft, supervision, project administration, methodology, funding acquisition, conceptualization.

## Supporting information


**Table S1:** Effects of the commercial biostimulants MU and SR compared with control (CTRL) on polyphenols, lycopene, glucose (Glc), fructose (Fru), sucrose, starch and total sugar index (TSI) (in mg g^−1^ FW) in processing tomato variety cv. “H1534.”
**Table S2:** Effects of the commercial biostimulants MU and SR compared with control (CTRL) on proteins (in mg g^−1^ FW) and free amino acids (in μmol g^−1^ FW) in processing tomato cv. “H1534” in processing tomato variety “H1534.”

## Data Availability

The data that support the findings of this study are already contained within this article or available in the Supporting Information S1.
