# Supplementary material for: Processing Tomato Responses to Plant‐Based Biostimulants Are Modulated by Environmental Conditions
Source: Physiol Plant. 2025 Aug 10;177(4):e70450. doi: 10.1111/ppl.70450 (PMC12336635; doi:10.1111/ppl.70450)
Supplement: Supplementary file 1 — Table S1: Effects of the commercial biostimulants MU and SR compared with control (CTRL) on polyphenols, lycopene, glucose (Glc), fructose (Fru), sucrose, starch and total sugar index (TSI) (in mg g−1 FW) in processing tomato variety cv. “H1534.” Table S2: Effects of the commercial biostimulants MU and SR compared with control (CTRL) on proteins (in mg g−1 FW) and free amino acids (in μmol g−1 FW) in processing tomato cv. “H1534” in processing tomato variety “H1534.” [file PPL-177-e70450-s001.pdf]

SUPPLEMENTARY TABLES

Processing Tomato Responses to Plant-Based Biostimulants Are Modulated by Environmental Conditions

Giovanna Marta Fusco[1,†] https://orcid.org/0009-0004-0268-4677**, Andrea Burato**[2,3,†] https://orcid.org/0000-0001-5425-5170**, Alfonso Pentangelo**[3]**, Petronia Carillo**[1*] https://orcid.org/0000-0003-3723-0398**, Mario Parisi**[3] https://orcid.org/0000-0001-5550-5135

[1]Department of Environmental, Biological and Pharmaceutical Sciences and Technologies, University of Campania "Luigi Vanvitelli", Via Vivaldi 43, 81100 Caserta, Italy
[2]School of Agricultural, Forest, Food and Environmental Sciences (SAFE), University of Basilicata, Viale dell'Ateneo Lucano 10, 85100 Potenza, Italy
[3]CREA Research Centre for Vegetable and Ornamental Crops, Council for Agricultural Research and Economics, Via Cavalleggeri 51, 84098 Pontecagnano, Italy
[†]These authors contributed equally to this work.

***Correspondence:**
Petronia Carillo, Email: petronia.carillo@unicampania.it

Table S1. Effects of the commercial biostimulants MU and SR compared to control (CTRL) on polyphenols, lycopene, glucose (Glc), fructose (Fru), sucrose, starch and total sugar index (TSI) (in mg g^{-1} FW) in processing tomato variety cv 'H1534'.

	**Polyphenols**		**Lycopene**		**Glucose**		**Fructose**		**Glc/Fru**	**Sucrose**		**Starch**	**TSI**	
Treatment (T)														
CTRL	0.33 ± 0.01	a	8.05 ± 0.20		12.38 ± 0.86		9.31 ± 0.75		9.30 ± 0.74	0.79 ± 0.05		4.34 ± 0.33	26.43 ± 1.88	
MU	0.42 ± 0.03	a	5.86 ± 1.03		12.50 ± 0.99		9.72 ± 1.01		9.72 ± 1.01	0.72 ± 0.12		3.49 ± 0.52	26.87 ± 2.37	
SR	0.35 ± 0.04	ab	6.65 ± 0.50		12.16 ± 0.30		8.87 ± 0.23		8.87 ± 0.22	0.69 ± 0.05		3.68 ± 0.11	25.67 ± 0.84	
Year (Y)														
2019	0.40 ± 0.01	a	9.64 ± 0.78	a	13.23 ± 0.66	a	9.96 ± 0.72	a	9.96 ± 0.72	0.93 ± 0.08	a	3.82 ± 0.27	28.35 ± 1.72	a
2020	0.33 ± 0.04	b	4.07 ± 0.38	b	11.46 ± 0.77	b	8.64 ± 0.60	b	8.64 ± 0.60	0.53 ± 0.06	b	3.85 ± 0.37	24.29 ± 1.67	b
TxY														
CTRL 2019	0.36 ± 0.01		10.31 ± 0.31	a	13.37 ± 0.48		9.72 ± 0.21		9.72 ± 0.20	0.87 ± 0.05	a	3.96 ± 0.34	28.31 ± 0.89	
MU 2019	0.44 ± 0.02		9.11 ± 1.81	ab	13.32 ± 1.17		10.75 ± 1.61		10.75 ± 1.61	0.98 ± 0.15	a	3.81 ± 0.39	29.14 ± 3.12	
SR 2019	0.41 ± 0.01		9.50 ± 0.22	ab	13.01 ± 0.33		9.40 ± 0.35		9.40 ± 0.35	0.94 ± 0.06	a	3.68 ± 0.07	27.60 ± 1.16	
CTRL 2020	0.30 ± 0.01		5.80 ± 0.08	ab	11.39 ± 1.24		8.89 ± 1.29		8.89 ± 1.28	0.71 ± 0.05	ab	4.71 ± 0.32	24.55 ± 2.86	
MU 2020	0.40 ± 0.04		2.61 ± 0.26	b	11.68 ± 0.81		8.69 ± 0.42		8.68 ± 0.41	0.46 ± 0.09	b	3.16 ± 0.64	24.59 ± 1.63	
SR 2020	0.29 ± 0.07		3.80 ± 0.79	ab	11.31 ± 0.26		8.34 ± 0.10		8.34 ± 0.10	0.43 ± 0.05	b	3.68 ± 0.16	23.74 ± 0.52	
Significance														
Treatment (T)	*		ns		ns		ns		ns	ns		ns	ns	
Year (Y)	*		***		**		*		ns	***		ns	*	
TxY	ns		***		ns		ns		ns	**		ns	ns	

All data are presented as mean ± standard error, n = 3. Different letters indicate significant mean differences according to Tukey HSD test ($p < 0.05$). *, ** and *** denote significant effects at $p \leq 0.05$, $p \leq 0.01$ and $p \leq 0.001$, respectively.

Table S2. Effects of the commercial biostimulants MU and SR compared to control (CTRL) on proteins (in mg g−1 FW) and free amino acids (in µmol g^{-1} FW) in processing tomato cv 'H1534'. in processing tomato variety 'H1534'.

	Treatment (T)						Year (Y)				TxY												Significance		
	CTRL		MU		SR		2019		2020		CTRL 2019		MU 2019		SR 2019		CTRL 2020		MU 2020		SR 2020		T	Y	TxY
Proteins	1.59 ± 0.18		1.41 ± 0.14		1.54 ± 0.05		1.56 ± 0.11		1.46 ± 0.14		1.32 ± 0.17	b	1.51 ± 0.13	ab	1.85 ± 0.03	a	1.85 ± 0.20	a	1.31 ± 0.15	b	1.22 ± 0.07	b	ns	ns	*
Ala	2.67 ± 0.28		2.80 ± 0.31		2.61 ± 0.23		1.89 ± 0.19	b	3.50 ± 0.36	a	1.43 ± 0.08	b	1.72 ± 0.13	b	2.51 ± 0.35	ab	3.92 ± 0.47	a	3.88 ± 0.48	a	2.71 ± 0.12	ab	ns	***	***
Arg	0.64 ± 0.07	a	1.73 ± 0.20	a	0.86 ± 0.09	ab	0.67 ± 0.06	b	1.48 ± 0.18	a	0.50 ± 0.06	b	0.68 ± 0.10	b	0.83 ± 0.04	b	0.77 ± 0.08	b	2.79 ± 0.30	a	0.89 ± 0.14	b	*	*	***
Asn	6.82 ± 0.92		8.04 ± 1.17		6.98 ± 0.20		7.01 ± 0.77		7.55 ± 0.76		5.64 ± 0.99		7.23 ± 1.08		8.17 ± 0.24		8.00 ± 0.85		8.86 ± 1.27		5.79 ± 0.17		ns	ns	ns
Asp	4.63 ± 0.71		6.10 ± 0.80		6.01 ± 0.35		5.57 ± 0.57		5.65 ± 0.67		4.28 ± 0.79		5.85 ± 0.85		6.58 ± 0.05		4.98 ± 0.63		6.35 ± 0.74		5.61 ± 0.64		ns	ns	ns
GABA	8.20 ± 0.31		11.10 ± 0.51		10.75 ± 0.11		7.92 ± 0.26	b	12.12 ± 0.37	a	6.08 ± 0.34	b	7.56 ± 0.39	b	10.12 ± 0.07	ab	10.33 ± 0.32	ab	14.65 ± 0.64	a	11.37 ± 0.16	ab	ns	**	*
Gln	12.55 ± 2.18		16.50 ± 1.80		16.00 ± 1.71		12.35 ± 1.14	b	17.69 ± 2.66	a	9.98 ± 1.61		12.38 ± 1.55		14.69 ± 0.25		15.12 ± 2.75		20.63 ± 2.04		17.31 ± 3.17		ns	*	ns
Glu	16.25 ± 2.63		21.30 ± 3.35		20.70 ± 0.82		22.39 ± 2.51	a	16.44 ± 2.02	b	17.34 ± 3.04		23.96 ± 2.43		25.88 ± 0.08		15.17 ± 2.22		18.63 ± 2.27		15.51 ± 1.57		ns	*	ns
Gly	0.33 ± 0.04		0.47 ± 0.07		0.44 ± 0.06		0.32 ± 0.03	b	0.50 ± 0.08	a	0.25 ± 0.03	b	0.30 ± 0.02	b	0.41 ± 0.03	ab	0.42 ± 0.05	ab	0.63 ± 0.11	a	0.46 ± 0.08	ab	ns	**	*
His	0.74 ± 0.11	ab	1.18 ± 0.16	a	1.11 ± 0.07	b	0.96 ± 0.10		1.06 ± 0.13		0.74 ± 0.13	ab	1.02 ± 0.16	ab	1.11 ± 0.02	ab	0.73 ± 0.10	b	1.33 ± 0.17	a	1.12 ± 0.12	ab	**	ns	*
Ile	0.33 ± 0.05		0.38 ± 0.02		0.39 ± 0.04		0.31 ± 0.02	b	0.43 ± 0.05	a	0.25 ± 0.03		0.31 ± 0.03		0.37 ± 0.01		0.41 ± 0.06		0.44 ± 0.01		0.42 ± 0.07		ns	**	ns
Leu	0.39 ± 0.04		0.51 ± 0.03		0.51 ± 0.03		0.37 ± 0.02	b	0.56 ± 0.05	a	0.32 ± 0.03	c	0.38 ± 0.03	c	0.43 ± 0.01	bc	0.46 ± 0.06	bc	0.64 ± 0.03	a	0.59 ± 0.05	ba	ns	***	***
MEA	0.35 ± 0.01		0.30 ± 0.01		0.29 ± 0.01		0.31 ± 0.01		0.32 ± 0.02		0.28 ± 0.02	a	0.31 ± 0.01	b	0.32 ± 0.01	b	0.41 ± 0.01	a	0.28 ± 0.02	b	0.26 ± 0.02	b	ns	ns	***
Orn	0.16 ± 0.01		0.20 ± 0.01		0.17 ± 0.01		0.13 ± 0.01	b	0.22 ± 0.01	a	0.12 ± 0.01	c	0.14 ± 0.01	c	0.13 ± 0.01	c	0.19 ± 0.01	b	0.25 ± 0.01	a	0.21 ± 0.01	ba	ns	***	***
Phe	0.63 ± 0.09	b	0.93 ± 0.14	a	0.87 ± 0.05	ab	0.75 ± 0.07		0.87 ± 0.11		0.55 ± 0.08		0.80 ± 0.14		0.87 ± 0.01		0.72 ± 0.09		1.03 ± 0.15		0.87± 0.10		*	ns	ns
Pro	0.76 ± 0.03		0.54 ± 0.08		0.50 ± 0.03		0.47 ± 0.03	b	0.73 ± 0.06	a	0.52 ± 0.03	b	0.48 ± 0.05	b	0.42 ± 0.02	b	0.99 ± 0.02	a	0.60 ± 0.11	b	0.59 ± 0.05	b	ns	**	***
Ser	1.29 ± 0.23		1.69 ± 0.18		1.44 ± 0.06		1.11 ± 0.10	a	1.84 ± 0.22	b	0.89 ± 0.12	a	1.04 ± 0.09	a	1.41 ± 0.09	a	1.68 ± 0.33	ab	2.34 ± 0.28	b	1.48 ± 0.04	ab	ns	**	**
Trp	0.27 ± 0.03		0.34 ± 0.05		0.30 ± 0.03		0.23 ± 0.03	a	0.38 ± 0.04	b	0.18 ± 0.02	a	0.24 ± 0.04	ac	0.28 ± 0.02	ab	0.37 ± 0.04	bc	0.44 ± 0.06	b	0.33 ± 0.03	ab	ns	***	**
Tyr	0.66 ± 0.11		0.90 ± 0.10		0.87 ± 0.12		0.60 ± 0.06	a	1.03 ± 0.16	b	0.49 ± 0.07	a	0.61 ± 0.09	ab	0.68 ± 0.01	ab	0.83 ± 0.14	ab	1.20 ± 0.11	b	1.07 ± 0.23	ab	ns	***	*
Val	0.29 ± 0.05		0.34 ± 0.02		0.36 ± 0.05		0.24 ± 0.02	a	0.41 ± 0.06	b	0.19 ± 0.02	a	0.24 ± 0.02	ab	0.30 ± 0.01	ab	0.39 ± 0.08	ab	0.43 ± 0.02	b	0.42 ± 0.08	b	ns	***	*
Total AA	58.64 ± 8.96		80.10 ± 11.90		73.76 ± 3.91		64.39 ± 6.33		77.27 ± 10.19		50.62 ± 8.12		66.12 ± 9.56		76.44 ± 1.30		66.67 ± 9.81		94.08 ± 14.24		71.08 ± 6.52		ns	ns	ns
BCAA	1.01 ± 0.14		1.22 ± 0.07		1.26 ± 0.11		0.93 ± 0.06	a	1.40 ± 0.15	b	0.76 ± 0.08	a	0.93 ± 0.07	ab	1.10 ± 0.03	ab	1.27 ± 0.20	ab	1.52 ± 0.06	b	1.43 ± 0.20	b	ns	***	**
Essential AA	3.33 ± 0.48		8.42 ± 2.98		6.05 ± 0.31		3.65 ± 0.34	a	8.22 ± 2.18	b	2.82 ± 0.40		3.84 ± 0.53		4.28 ± 0.08		3.84 ± 0.56		12.99 ± 5.43		7.82 ± 0.54		ns	*	ns

All data are presented as mean ± standard error, n = 3. Different letters indicate significant mean differences according to Tukey HSD test ($p < 0.05$). *, ** and *** denote significant effects at $p \leq 0.05$, $p \leq 0.01$ and $p \leq 0.001$, respectively.
